# Serum Alkaline Phosphatase Levels Predict Infection-Related Mortality and Hospitalization in Peritoneal Dialysis Patients

**DOI:** 10.1371/journal.pone.0157361

**Published:** 2016-06-16

**Authors:** Seun Deuk Hwang, Su-Hyun Kim, Young Ok Kim, Dong Chan Jin, Ho Chul Song, Euy Jin Choi, Yong-Lim Kim, Yon-Su Kim, Shin-Wook Kang, Nam-Ho Kim, Chul Woo Yang, Yong Kyun Kim

**Affiliations:** 1 Department of Internal Medicine, College of Medicine, The Catholic University of Korea, Seoul, Korea; 2 Department of Internal Medicine, College of Medicine, Chung-Ang University, Seoul, Korea; 3 Department of Internal Medicine, School of Medicine, Kyungpook National University, Daegu, Korea; 4 Department of Internal Medicine, College of Medicine, Seoul National University, Seoul, Korea; 5 Department of Internal Medicine, College of Medicine, Yonsei University, Seoul, Korea; 6 Department of Internal Medicine, Chonnam National University Medical School, Gwangju, Korea; 7 Cell Death Disease Research Center, The Catholic University of Korea, Seoul, Korea; Hospital Universitario de La Princesa, SPAIN

## Abstract

**Background:**

Serum alkaline phosphatase (ALP) levels have been reported to be associated with all-cause and cardiovascular mortality in peritoneal dialysis (PD) patients. However, it is unclear whether serum ALP levels predict infection-related clinical outcomes in PD patients. The aim of this study was to determine the relationships between serum ALP levels, infection-related mortality and hospitalization in PD patients.

**Methods:**

PD patients from the Clinical Research Center registry for end-stage renal disease, a multicenter prospective observational cohort study in Korea, were included in the present study. Patients were categorized into three groups by serum ALP tertiles as follows: Tertile 1, ALP <78 U/L; Tertile 2, ALP = 78–155 U/L; Tertile 3, ALP >155 U/L. Tertile 1 was used as the reference category. The primary outcomes were infection-related mortality and hospitalization.

**Results:**

A total of 1,455 PD patients were included. The median follow-up period was 32 months. The most common cause of infection-related mortality and hospitalization was PD-related peritonitis. Multivariate Cox regression analyses showed that patients in the highest tertiles of serum ALP levels were at higher risk of infection-related mortality (HR 2.29, 95% CI, 1.42–5.21, P = 0.008) after adjustment for clinical variables. Higher tertiles of serum ALP levels were associated with higher risk of infection-related hospitalization (Tertile 2: HR 1.56, 95% CI, 1.18–2.19, P = 0.009, tertile 3: HR 1.34, 95% CI, 1.03–2.62, P = 0.031).

**Conclusions:**

Our data showed that elevated serum ALP levels were independently associated with a higher risk of infection-related mortality and hospitalization in PD patients.

## Introduction

Alkaline phosphatase (ALP) is a hydrolase enzyme that removes phosphate from nucleotides and proteins and is used as an early differentiation marker of osteoblast and osteoblastic activity [1]. In addition to a marker of bone metabolism, experimental studies demonstrated that ALP is upregulated in vessels under uremic conditions and involved in vascular calcification by hydrolysis and inactivation of pyrophosphate, a potent vascular calcification inhibitor [2]. Epidemiologic studies have reported that increased serum total ALP levels are associated with higher all-cause or cardiovascular mortality and morbidity in hemodialysis (HD) patients [3–5].

Peritoneal dialysis (PD) patients may have different bone metabolism from HD patients [6], and are observed to have different patterns of all-cause mortality and cause-specific mortality from HD patients [7,8]. All-cause mortality is higher in PD patients, and Korean PD patients had higher risk of infection-related mortality than HD patients [9].

However, there are few biomarkers predicting infection-related clinical outcomes in PD patients. A previous study demonstrated that bone cell differentiation interplays with B cell differentiation, which indicates that bone metabolism may influence the innate immune system [10]. Furthermore, the previous retrospective studies reported that chronic kidney disease (CKD)-mineral and bone disorders (MBD), an established risk factor for vascular calcification in CKD patients, was reported to be associated with short-term adverse outcomes of PD-related peritonitis in PD patients [11,12].

Therefore, considering serum ALP levels as an early marker of bone cell differentiation as well as a biomarker of CKD-MBD, we postulated that serum ALP levels may be associated with infection-related clinical outcomes in PD patients. However, limited data are available regarding the relationship between serum ALP levels and infection-related clinical outcomes in PD patients.

The aim of this study was to determine whether serum ALP levels are associated with infection-related mortality and hospitalization in PD patients through a multicenter prospective observational cohort study in Korea.

## Materials and Methods

### Study population

All patients in this study participated in the Clinical Research Center (CRC) registry for end-stage renal disease (ESRD). This study is an observational prospective cohort study of patients with ESRD from 31 centers in Korea. The study was performed from April 2009 to April 2015, and included adult (>18 years of age) dialysis patients. A total of 1,801 patients with PD treatment were enrolled in this study. For the present study, patients were excluded for incomplete or missing values that made information unclear and covariate outlier data (n = 346). Finally, 1,455 patients were enrolled. The CRC for ESRD was approved by the medical ethics committees of the participating hospitals and informed consent was obtained from all patients before inclusion.

### Ethics

This study was approved by the institutional review board at each center (The Catholic University of Korea, Bucheon St. Mary's Hospital; The Catholic University of Korea, Incheon St. Mary's Hospital; The Catholic University of Korea, Seoul St. Mary's Hospital; The Catholic University of Korea, St. Mary's Hospital; The Catholic University of Korea, St. Vincent's Hospital; The Catholic University of Korea, Uijeongbu St. Mary's Hospital; Cheju Halla General Hospital; Chonbuk National University Hospital; Chonnam National University Hospital; Chung-Ang University Medical Center; Chungbuk National University Hospital; Chungnam National University Hospital; Dong-A University Medical Center; Ehwa Womens University Medical Center; Fatima Hospital, Daegu; Gachon University Gil Medical Center; Inje University Pusan Paik Hospital; Kyungpook National University Hospital; Kwandong University College of Medicine, Myongji Hospital; National Health Insurance Corporation Ilsan Hospital; National Medical Center; Pusan National University Hospital; Samsung Medical Center, Seoul; Seoul Metropolitan Government, Seoul National University, Boramae Medical Center; Seoul National University Hospital; Seoul National University, Bundang Hospital; Yeungnam University Medical Center; Yonsei University, Severance Hospital; Yonsei University, Gangnam Severance Hospital; Ulsan University Hospital; Wonju Christian Hospital [in alphabetical order]) and performed in accordance to the Declaration of Helsinki. Written informed consent was obtained from all patients.

### Data collection

Demographic and clinical data including age, sex, height, weight, body mass index (BMI), co-morbidities, laboratory investigations and therapeutic characteristics were recorded at the time of enrollment. Assessments of dialysis characteristics and measurements of health were performed every 6 months until follow-up was complete. Cardiovascular disease was defined as the presence of coronary artery disease, congestive heart failure, peripheral vascular disease or cerebrovascular disease. Serum hemoglobin, albumin, aspartate aminotransferase (AST), alanine aminotransferase (ALT), calcium, phosphorus, intact parathyroid hormone (PTH), total cholesterol (TC), triglyceride (TG), uric acid and high-sensitivity C-reactive protein (hsCRP) were determined from blood samples. Serum ALP levels were measured at 30°C using fasting blood samples and optimal pH was determined to be 9–10 by a standard method of the International Federation of Clinical Chemistry (IFCC) [13] Patients were categorized into three groups by tertiles of serum ALP levels as follows: Tertile 1, ALP < 78 U/L; Tertile 2, ALP = 78–155 U/L; Tertile 3, ALP > 155 U/L.

### Outcomes

The primary outcomes were infection-related mortality and hospitalization. All patients were followed until death or the end of the study, with censoring of data at the time that a patient underwent renal transplantation or was lost to follow-up because of patient refusal of further participation or transfer to a nonparticipating hospital. For each death and hospitalization, the principal investigators of the clinical center completed a form including the cause of death or hospitalization according to the CRC for ESRD study classification.

### Statistical Analyses

Data for continuous variables with normal distributions are presented as mean ± SD and those without normal distribution are presented as the median with ranges as appropriate for the type of variable. Student’s t-test, the Mann–Whitney test, one-way ANOVA, or the Kruskal-Wallis test were used, as appropriate, to determine differences in continuous variables. Categorical variables are presented as percentages. The Pearson’s chi-square test or Fisher’s exact test was used to determine the differences in categorical variables.

Absolute mortality rates were calculated per 100 person-years of follow-up. The survival curves were estimated using the Kaplan-Meier method and compared using the log-rank test. The Cox proportional hazard regression model was used to calculate the hazard ratio (HR) with 95% confidence interval (CI) for infection-related mortality and hospitalization, using tertile 1 as the reference category. The Cox models were adjusted for significant or nearly significant (p < 0.1) predictors of all-cause mortality in univariate Cox regression analysis including age, hemoglobin levels, serum levels of ferritin, intact PTH, albumin, ALP and duration of dialysis. For adequate control of confounders, important covariates known to be influential based on prior studies and clinical insight, such as sex, diabetes mellitus, cardiovascular disease and serum hs-CRP levels were retained in the multivariate Cox regression model regardless of their statistical significance. Analyses were adjusted for potential confounders using two models. Model 1 was adjusted for age and sex. Model 2 was adjusted for age, sex, diabetes mellitus, cardiovascular diseases, hemoglobin levels, serum levels of ferritin, hs-CRP, albumin, AST, ALT, TC, calcium and phosphate. A value of P < 0.05 was considered statistically significant. All statistical analyses were performed using SPSS 16.0 software (Chicago, IL, USA).

## Results

### Patient Characteristics

A total of 1,455 PD patients were included. The median serum ALP level was 104 IU/L (interquartile range, 69–191 IU/L). Baseline characteristics of the study population by tertiles of serum ALP levels are shown in Table 1. Patients with higher ALP levels were older than those with lower levels. Patients with higher ALP levels had longer duration of dialysis, higher hemoglobin levels, higher serum levels of ALT, TC, ferritin, intact PTH and anti-HBs antigen (Ag) positivity and lower serum levels of phosphorous. There were no differences in sex, body mass index (BMI), primary renal diseases, the prevalence of diabetes and previous cardiovascular disease, modified Charlson comorbidity index, systolic blood pressure (BP), diastolic BP, serum levels of protein, albumin, AST, calcium, uric acid, hsCRP, or anti-HCV antibody (Ab) positivity among groups.

**Table 1:** Baseline characteristics of patients according to baseline serum ALP tertile.

		Overall	ALP (IU/L)			P
			Tertile 1<78.0 U/L	Tertile 278–155 U/L	Tertile 3>155 U/L	
**Number of patients**		1455	490	484	481	
**Age (years)**		52±12	51±12	52±13	53±11	0.039
**Male, n (%)**		833 (57.2)	303 (61.8)	263 (54.1)	274 (55.7)	0.056
**Body mass index (kg/m**^**2**^**)**		23.3±3.2	23.1±3.1	23.3±3.3	23.4±3.3	0.472
**Primary renal disease, n (%)**						0.051
	**Diabetes mellitus**	705 (48.5)	227 (46.3)	235 (48.6)	243 (50.5)	
	**Hypertension**	332 (22.8)	103 (21.0)	108 (22.3)	121 (25.2)	
	**Glomerulonephritis**	259 (17.8)	90 (18.4)	91 (18.8)	78 (16.2)	
	**Other**	159 (10.9)	70 (14.3)	50 (10.3)	39 (8.1)	
**Co-morbidity, n (%)**						
	**Diabetes mellitus**	814 (57.0)	280 (59.3)	283 (59.1)	251 (52.7)	0.067
	**Cardiovascular disease**	216 (14.9)	104 (21.4)	74 (15.3)	38 (7.9)	0.093
**Modified CCI**		4.3±2.2	4.1±2.2	4.3±2.2	4.4±2.0	0.123
**Duration of dialysis, months**		36 (14–65)	28 (11–54)	40 (17–67)	41 (17–67)	0.027
**Systolic BP, mmHg**		134±21	134±20	133±21	134±22	0.811
**Diastolic BP, mmHg**		79±13	79±12	78±13	80±13	0.210
**Hemoglobin, g/dl**		10.0±1.6	9.9±1.7	9.9±1.6	10.3±1.5	0.013
**Serum protein, g/dl**		6.3±0.7	6.3±0.7	6.4±0.8	6.4±0.7	0.058
**Serum albumin, g/dl**		3.5±0.5	3.6±0.5	3.5±0.6	3.5±0.5	0.363
**Serum AST, IU/L**		20.3±17.9	19.5±23.6	20.8±14.6	20.6±13.6	0.475
**Serum ALT, IU/L**		19.9±19.2	17.8±14.2	21.0±24.1	20.9±17.7	0.015
**Serum calcium, mg/dl**		8.4±0.9	8.3±0.9	8.45±1.01	8.4±0.9	0.503
**Serum phosphorus, mg/dl**		5.1±1.6	5.3±1.6	5.2±1.7	5.0±1.5	0.003
**Serum TC, mg/dl**		171.8±44.3	169.3±46.5	169.2±44.1	177.4±41.7	0.014
**Serum uric acid, mg/dl**		7.3±1.9	7.52±2.0	7.24±2.0	7.2±1.6	0.253
**Serum hsCRP, mg/dl**		0.1 (0.02–0.47)	0.1 (0.02–0.52)	0.13 (0.03–0.59)	0.07 (0.01–0.32)	0.137
**Serum ferritin, ng/ml**		185 (88–370)	169 (81–333)	176 (85–352)	214 (99–413)	0.021
**Serum intact PTH**		204 (101–352)	168 (86–294)	224 (121–382)	229 (97–368)	0.014
**HBs Ag (+), n (%)**		536 (36.8)	143 (29.2)	180 (37.2)	213 (44.3)	<0.001
**HCV Ab (+), n (%)**		32 (3.7)	11 (3.4)	8 (2.7)	13 (5.3)	0.275

Values for continuous variables given as means ± standard deviation and variables not normally distributed given as median and interquartile range; values for categorical variables given as numbers (percentages). ALP, alkaline phosphatase; CCI, Charlson comorbidity index; BP, blood pressure; AST, aspartate aminotransferase; ALT, alanine aminotransferase; PTH, parathyroid hormone; TC, total cholesterol; hsCRP, high sensitivity C-Reactive Protein; SGA, Subjective Global Assessment.

### Determinants of serum ALP levels

Linear regression analysis indicated that serum ALP levels were associated with clinical variables (Table 2). In the univariate model, serum ALP levels were positively correlated with duration of dialysis, systolic BP, hemoglobin levels, serum levels of TC, ferritin and intact PTH and HBs Ag positivity and negatively correlated with serum phosphorus levels. In a stepwise multiple linear regression model including all univariate correlates of serum ALP levels, serum phosphorus level (β = -0.098, P = 0.035), serum intact PTH (β = 0.252, P<0.001) and HBs Ag positivity (β = 0.366, P<0.001) were independently correlated with serum ALP levels.

**Table 2:** Univariate linear regression analysis of serum ALP levels and clinical variables.

	β	P value
**Age (years)**	0.051	0.050
**Body mass index (kg/m**^**2**^**)**	0.000	0.993
**Diabetes mellitus**	0.025	0.343
**Duration of dialysis, months**	0.131	0.001
**Systolic BP (mmHg)**	-0.017	0.536
**Diastolic BP (mmHg)**	0.001	0.973
**Hemoglobin (g/dl)**	0.105	0.001
**Protein (g/dl)**	0.038	0.151
**Albumin (g/dl)**	-0.019	0.468
**Serum AST (IU/L)**	0.034	0.197
**Serum ALT (IU/L)**	0.049	0.061
**Calcium (mg/dl)**	0.027	0.298
**Phosphorus (mg/dl)**	-0.082	0.002
**TC (mg/dl)**	0.071	0.009
**Uric acid (mg/dl)**	-0.041	0.125
**CRP**	-0.044	0.139
**Ferritin (ng/mL)**	0.110	0.001
**Intact PTH**	0.121	0.001
**HBs Ag (+)**	0.084	0.002
**HCV Ab (+)**	0.017	0.642

BP, blood pressure; AST, aspartate aminotransferase; ALT, alanine aminotransferase; PTH, parathyroid hormone; TC, total cholesterol; TG, triglyceride; hsCRP, high-sensitivity C-reactive protein.; SGA, Subjective Global Assessment. Multiple regression analysis model including age, sex, duration of dialysis therapy, serum levels of albumin, hemoglobin, ALT, total cholesterol, intact PTH, ferritin, calcium and phosphate.

### Associations between serum ALP levels and infection-related mortality

The median follow-up period was 32 months (interquartile range, 15–49 months). During the follow-up period, 188 patients left the study. The reasons for censoring included kidney transplantation (n = 91), transfer to a nonparticipating hospital (n = 65), refusal of further participation (n = 11) and other (n = 21). Cardiovascular disease was the leading cause of death (42% of all deaths), followed by infection-related disease (39% of all deaths).

**Fig 1:**
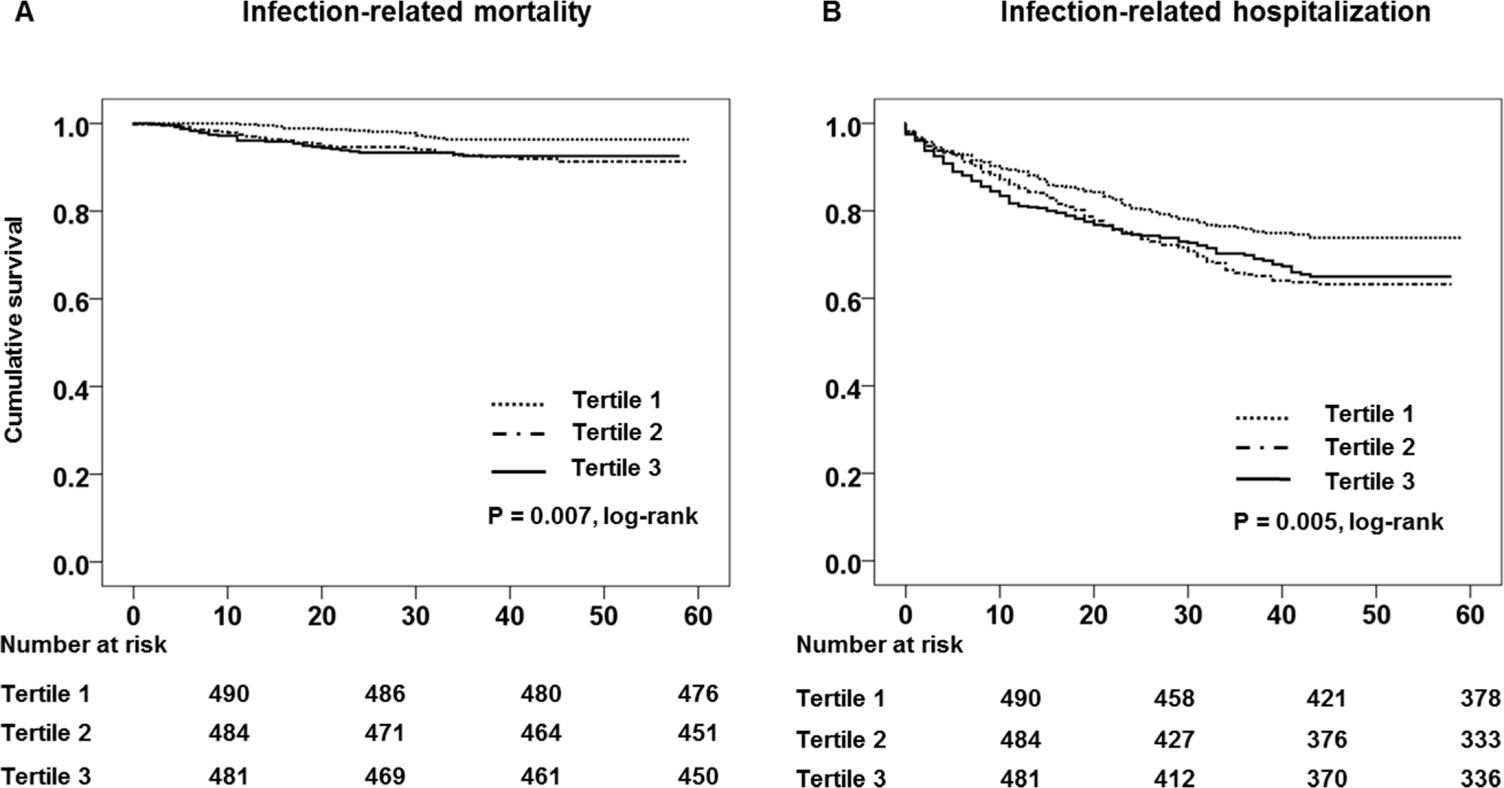
Kaplan-Meier survival curves for (A) infection-related mortality and (B) infection-related hospitalization.

There were 141 infection-related deaths during the follow-up period. The absolute infection-related mortality rate was 3.6 deaths per 100 person-years. Table 3 shows the distribution of causative diseases for infection-related deaths during the follow-up period. PD-related peritonitis was the most common causes of infection-related deaths (43% of infection-related death). Death from PD-related peritonitis was more likely in the higher tertiles of serum ALP levels (p = 0.021). A Kaplan-Meier plot showed that infection-related mortality rate was significantly increased in patients with the higher tertiles of serum ALP levels (P = 0.007, log-rank test) (Fig 1A). Table 4 shows the results of univariate and multivariate Cox regression analysis for infection-related mortality. In the crude model, the hazard ratio (HR) of infection-related mortality of patients in tertile 2 of serum ALP levels was 2.46 (95% CI, 1.32–4.60, P = 0.005) and HR of tertile 3 was 2.42 (95% CI, 1.29–4.56, P = 0.006) using tertile 1 as the reference category. In multivariate Cox regression analysis, tertile 3 had a significantly independent higher risk for infection-related mortality than tertile 1 even after adjusting for demographics, laboratory data, and comorbid conditions (model 1: HR 2.47, 95% CI, 1.31–4.66, P = 0.005; model 2: HR 2.29, 95% CI, 1.42–5.21, P = 0.008).

**Table 3:** Causes of infection-related deaths and hospitalization by serum tertile.

	ALP (U/L)			P
	Tertile 1	Tertile 2	Tertile 3	
**Infection-related deaths**				
**PD-related peritonitis, n (%)**	5 (35.7)	15 (45.5)	14 (45.2)	0.021
**Sepsis, n (%)**	3 (21.4)	9 (27.3)	6 (19.4)	0.263
**Pneumonia, n (%)**	2 (14.3)	4 (12.1)	5 (16.1)	0.122
**UTI, n (%)**	1 (7.1)	1 (3.0)	3 (9.7)	0.435
**Others, n (%)**	3 (21.4)	4 (12.1)	3 (9.7)	0.351
**Total, n (%)**	14 (100)	33 (100)	31 (100)	
**Infection-related hospitalization**				
**PD-related peritonitis**	71(63.4)	106(70.2)	90(62.1)	0.292
**Sepsis, n (%)**	5(4.5)	13(8.6)	10(6.9)	0.439
**Pneumonia, n (%)**	5(4.5)	7(4.6)	7(4.8)	0.506
**UTI, n (%)**	4(3.6)	3(2.0)	6(4.1)	0.368
**Diabetic foot infection, n (%)**	2(1.8)	1(0.7)	0	0.568
**Others, n (%)**	25(22.3)	21(13.9)	32(22.1)	0.634
**Total, n (%)**	112 (100)	151 (100)	145 (100)	

ALP, alkaline phosphatase.

**Table 4:** Univariate and multivariate Cox regression analysis for infection-related mortality and hospitalization serum ALP tertile.

		Crude model			Model 1			Model 2		
		HR	95% CI	P	HR	95% CI	P	HR	95% CI	P
**Infection-related mortality**										
	**Tertile 1**	1 (reference)			1 (reference)			1 (reference)		
	**Tertile 2**	2.46	1.32–4.60	0.005	2.53	1.35–4.75	0.004	1.88	0.95–3.81	0.072
	**Tertile 3**	2.42	1.29–4.56	0.006	2.47	1.31–4.66	0.005	2.29	1.42–5.21	0.008
**Infection-related hospitalization**										
	**Tertile 1**	1 (reference)			1 (reference)			1 (reference)		
	**Tertile 2**	1.45	1.13–1.85	0.003	1.43	1.19–1.82	0.004	1.53	1.18–2.19	0.006
	**Tertile 3**	1.40	1.10–1.79	0.007	1.36	1.06–1.74	0.013	1.34	1.03–2.62	0.031

ALP, alkaline phosphatase; HR, hazard ratio; CI, confidence interval; CV, cardiovascular. Model 1: Multivariate model including age and sex. Model 2: Multivariate model including model 1 + duration of dialysis therapy, diabetes mellitus, cardiovascular diseases, hemoglobin, serum levels of albumin, ALT, total cholesterol, intact PTH, ferritin, calcium and phosphate.

### Associations between serum ALP levels and infection-related hospitalization

A total of 899 hospitalization events occurred during the follow-up period. The most common cause of hospitalization was infection-related hospitalization (n = 408, 45%). Table 3 shows the distribution of causative diseases in patients with infection-related hospitalization during the follow-up period. The most common causative disease in patients with infection-related hospitalization was PD-related peritonitis (n = 267, 65.4%), and the distribution of causative diseases in patients with infection-related hospitalization was not different among the tertiles of serum ALP levels.

Fig 1B shows the Kaplan-Meier plot of infection-related hospitalization according to tertiles of serum GGT levels. The log rank test showed that the infection-related hospitalization rate was significantly increased in patients in higher tertiles of serum ALP levels (P = 0.005). Table 4 shows the results of Cox regression analyses for infection-related hospitalization. In multivariate Cox regression analysis, tertiles 2 and 3 had significantly higher independent risk of infection-related hospitalization than tertile 1 in model 1 (tertile 2: HR, 1.43, 95% CI, 1.19–1.82, P = 0.004 and tertile 3: HR, 1.36, 95% CI, 1.06–1.74, P = 0.013) and in model 2 (tertile 2: HR, 1.53, 95% CI, 1.18–2.19, P = 0.006 and tertile 3: HR, 1.34, 95% CI, 1.03–2.62, P = 0.031).

## Discussion

In this prospective observational study, we demonstrated that higher serum ALP levels were associated with increased infection-related mortality and hospitalization in PD patients independent of other markers of CKD-MBD, including serum calcium, phosphorus and intact PTH, and independent of liver disease. To our knowledge, this is the first multicenter prospective study demonstrating associations between serum ALP levels and infection-related mortality and hospitalization in PD patients.

Some large cohort studies have demonstrated associations between serum ALP levels and all-cause mortality and cardiovascular mortality in PD patients [14]. However, the relationships between serum ALP levels and non-cardiovascular mortality, especially infection-related mortality in PD patients, had not been established.

Our findings support the hypothesis that elevated ALP levels can affect infection-related clinical outcomes. Furthermore, because infection-related mortality is one of the most common causes of death in PD patients, our findings underscore the prognostic power of serum ALP level to predict non-cardiovascular clinical outcomes, especially infection-related mortality and hospitalization, in PD patients.

The mechanism underlying the association between ALP and infection-related clinical outcomes in PD patients remains unclear. However, some explanations can be proposed.

In response to infection, Toll-like receptors (TLRs), especially TLR4 on immune cells (ICs) play a critical role in pathogen recognition by binding to the lipopolysaccharide (LPS) derived from bacteria, which is the endotoxin as an important pathogen-associated molecular pattern causing sepsis through TLR4 signaling [15]. Subsequently, the inflammatory response is triggered by enhancing the transcription factor NF-kB (nuclear factor-kB) signaling, which results in the systemic secretion of TNF-α and other pro- and anti-inflammatory cytokines and recruiting neutrophils for bacteria removal. ALP interacts with the LPS-TLR4 pathway and can reduce LPS through the dephosphorylation of LPS [11,15]. Campbell et al demonstrated that the endogenous intestinal ALP induced by the proinflammatory Resolvin-E1 reduced the activity of NF-kB signaling induced by LPS, which resulted in the reduction of the release of pro-inflammatory cytokines, including TNF-α, interleukin 6, and LPS-binding protein [16]. Considering this relationship between ALP and infection, it may be cautiously postulated that the increased serum ALP levels may be a response of infection in patients. PD patients have high risk of infection due to the continuous peritoneal exposure to peritoneal dialysate and PD catheter [8]. In this study, PD patients had higher serum ALP levels without overt infection-related symptoms at enrollment had higher infection-related mortality and hospitalizations during the follow-up period. Therefore, we cautiously suggest that the higher serum ALP levels may be the biomarker of subclinical infection or inflammation, which may be susceptible to overt-infection such as PD-related peritonitis. To clarify this mechanism, further studies are needed.

Serum ALP is a biochemical marker of high-turnover bone disease and is used to monitor the CKD-MBD. CKD-MBD has been reported to be associated with the development of peritoneal calcifications in a PD patient [17] and the presence and severity of peritoneal calcification are associated with peritonitis [18]. Chuang et al reported that the failure of serum calcium, phosphorous and intact-PTH concentration to achieve KDIGO’s targets for CKD-MBD was related with incidence of peritonitis or exit-site infections in incident PD patients in their retrospective study [12]. Therefore, it may be postulated that peritoneal calcification in PD patients with CKD-MBD have higher risk of PD-related peritonitis and consequently serum ALP as a biomarker of CKD-MBD may predict PD-related peritonitis. However, in this study, serum ALP levels had independent prognostic value for infection-related clinical outcomes even after adjustment for markers of CKD-MBD, including serum calcium, phosphorus and intact PTH, which is compatible with the results of previous studies in HD and PD patients [4,11,14]. It may be due that serum ALP might be not only a biomarker of CKD-MBD but also, a prognostic factor for infection-related mortality and morbidity such as inflammation [14,19]. To determine this explanation, further studies are needed.

The Dialysis Outcomes and Practice Patterns Study (DOPPS) reported that higher serum ALP levels were related with lower serum albumin levels and higher infection-related mortality in HD patients [4]. It may suggest that high serum ALP levels reflect with poor nutritional status, which was susceptible to severe infections [4]. However, in this study, serum ALP levels were not associated with nutritional marker such as serum albumin levels and BMI, which suggest that elevated ALP levels may not reflect malnutrition status in PD patients different from HD patients.

One of the most interesting findings in our study is that the association between serum ALP levels and infection-related clinical outcomes was independent of liver diseases. Serum ALP levels are used in clinical practice as a biomarker of liver diseases [20]. Liver diseases such as hepatitis B and hepatitis C are prevalent and have been reported to be a risk factor for mortality and morbidity in PD patients [14,21]. To determine whether the association of serum ALP levels is dependent or independent of liver disease, we performed analyses that were adjusted for liver disease, including HBS Ag and anti-HCV Ab positivity, serum AST and ALT levels. Our results showed that serum ALP levels had independent prognostic value for infection-related clinical outcomes even after adjustment for liver disease, which is compatible with the results of previous studies in HD and PD patients [4,11,14].

Another interesting finding of our study is that high serum ALP levels were associated with a higher risk of peritonitis and peritonitis-related morbidity and mortality. PD-related peritonitis is one of the most common infectious complications in PD patients. In this study, PD-related peritonitis was the most common cause of death or hospitalization. Ye et al. previously reported that higher serum ALP was associated with higher risk of short-term adverse outcomes of PD-related peritonitis [11]. In this study, we demonstrated the prognostic power of serum ALP levels to predict not only short-term adverse outcomes but also peritonitis-related mortality. While considering that serum ALP levels are relatively easily measurable and typically used in clinical practice, our data suggest that serum ALP may be a useful biomarker of infection-related clinical outcomes, especially PD-related peritonitis.

The strengths of our study are the relatively large number of PD patients included and the multi-center design, which involved patients from 31 centers in Korea. And, we demonstrated the relationship between ALP and infection-related death or hospitalization.

Our study has some potential limitations. First, our study was an observational study rather than a randomized controlled trial. Second, despite the multicenter nature of the study, the cohort consisted solely of ethnic Korean patients. Thus it is uncertain whether our results can be generalized to other ethnic groups with ESRD. Third, serum ALP levels can change over time. However, repeated ALP measurements were not performed in this study. Therefore we were unable to determine the prognostic value of longitudinal changes of serum ALP levels on infection-related clinical outcomes.

In conclusion, we demonstrated that high serum ALP levels are an independent risk factor for infection-related mortality and hospitalization in PD patients. Our findings suggest that serum ALP levels are a useful biomarker for predicting infection-related clinical outcomes in PD patients.
